# Laminoplasty instead of laminectomy as a decompression method in posterior instrumented fusion for degenerative cervical kyphosis with stenosis

**DOI:** 10.1186/s13018-015-0280-y

**Published:** 2015-09-04

**Authors:** Kuang-Ting Yeh, Ru-Ping Lee, Ing-Ho Chen, Tzai-Chiu Yu, Kuan-Lin Liu, Cheng-Huan Peng, Jen-Hung Wang, Wen-Tien Wu

**Affiliations:** Institute of Medical Sciences, Tzu Chi University, No. 701, Zhongyang Rd., Sec. 3, Hualien, 97004 Taiwan; Department of Orthopedics, Hualien Tzu Chi Hospital, Buddhist Tzu Chi Medical Foundation, Hualien, 97002 Taiwan; School of Medicine, Tzu Chi University, Hualien, 97004 Taiwan; Department of Research, Hualien Tzu Chi Hospital, Buddhist Tzu Chi Medical Foundation, Hualien, 97002 Taiwan

**Keywords:** Degenerative cervical kyphosis with stenosis, Laminoplasty, Fusion bed, Posterior instrumented fusion, Perineural adhesion

## Abstract

**Background:**

Posterior laminectomy with instrumented fusion is a standard procedure for treating degenerative cervical kyphosis with stenosis (DCKS). Two major disadvantages of the surgery are adhesion of the dural membrane with significant disfiguring of cervical spine and a small fusion bed around the lateral mass. One of the advantages of laminoplasty over laminectomy is the protection of the dural membrane from adhesion through preservation of posterior bony elements. This study presents the surgical outcomes of laminoplasty, instead of laminectomy, as a decompression method applied in posterior instrumented fusion for DCKS.

**Methods:**

A consecutive single center series of twenty cases between 2008 and 2011 were retrospectively reviewed. They were diagnosed as DCKS and received anterior cervical fusion followed by expansive open door laminoplasty and lateral mass or pedicle screw instrumented fusion. We collected the functional scores and changes of cervical curvature on the basis of dynamic lateral films preoperatively and postoperatively. We used computed tomography scans and magnetic resonance imaging (MRI) to evaluate the status of fusion and decompression.

**Results:**

The mean age at the time of surgery was 67.6 ± 15.2 years. Half of the patients were older than 75 years. All functional scores and cervical lordotic curvatures markedly improved. No recurrence of spinal cord compression was caused by closure of opened laminae, according to MRI study that was conducted 12 months postoperatively. No pseudarthrosis or hardware loosening was observed 24 months postoperatively.

**Conclusion:**

The surgical aims for DCKS are adequate decompression, correction of kyphosis, and solid instrumented fusion. Laminoplasty applied in cervical fusion as a decompression method seems to lead to a favorable functional recovery and reduces the complications of perineural adhesion that typically occur after laminectomy. In addition, laminoplasty affords an additional fusion bed at the hinge side and this advantage benefits solid fusion mass formation for the patients who suffered from DCKS.

## Introduction

Posterior decompression with instrumented fusion is a standard method for treating degenerative cervical kyphosis with stenotic myelopathy [1]. Adequate decompression and osteophyte removal through laminectomy, solid fusion through bilateral posterolateral fusion with bone grafts, and instrumentation with a lateral mass or pedicle screw system are essential steps to achieve favorable surgical results [2]. For fixed kyphotic deformity, which cannot be corrected by using a posterior approach, additional anterior cervical decompression fusion is required and is achieved through resection of an anterior longitudinal ligament, removal of anterior compression, and restoration of the intervertebral disk height [3]. However, the two major disadvantages of posterior laminectomy and instrumented fusion are adhesion of the dural membrane and a small fusion bed of the posterolateral cervical spine, which can cause postoperative neck pain, subsequent neurologic deterioration, and increased risk of iatrogenic spinal cord injury as well as difficulty in performing future revision surgeries [4, 5].

Laminoplasty is used to decompress the cervical spinal cord and is generally applied in multilevel cervical stenosis without local kyphotic deformity and instability [6]. The superiority of laminoplasty over laminectomy with fusion can be attributed to the protection of the dural membrane from adhesion through the preservation of posterior bony elements in multilevel cervical myelopathy [7]. For patients with degenerative cervical kyphosis with stenosis (DCKS) that require posterior instrumented fusion, posterior decompression with laminoplasty is not only no more complex than laminectomy but also more beneficial in preventing the dura from adhesion and providing an additional fusion bed through the preservation of posterior bony structures.

In our institute, the number of total spinal surgeries is about 720 in thoracolumbar spine and 300 in cervical spine yearly in average. Cervical surgeries include expansive open door laminoplasty (EOLP), anterior cervical discectomy and fusion (ACDF), anterior cervical corpectomy and fusion (ACCF), laminoplasty with adjunct anterior or posterior short segment fusion, occipitocervical fusion, C1-2 posterior fusion, and long-level cervical instrumented fusion with or without anterior segmental fusion. Most of our posterior procedures of cervical spine include laminoplasty as a decompression method for spinal cord.

In consideration of the advantages of laminoplasty, which can resolve the complications of posterior instrumented fusion with laminectomy, for the first time, this study presents the postoperative (24 months) surgical outcomes of circumferential corrective fusion surgery and laminoplasty for DCKS. We sought to understand the availability and effectiveness of laminoplasty applied in cervical posterior decompression and instrumented fusion.

## Material and methods

A consecutive single-center series of 20 cases between 2008 and 2011 were retrospectively reviewed. The inclusion criteria were that patients suffered from the diagnosis of DCKS and underwent one-stage anterior and posterior decompression and fusion procedure.

The indication of this surgery is that the patients suffered from myelopathic symptoms and were diagnosed as DCKS. The diagnostic criteria of DCKS include the following: (1) marked degenerative cervical spondylosis with fixed cervical kyphotic deformity through dynamic plain films; (2) C2-7 stenosis with spinal cord compression with local kyphotic deformity through magnetic resonance imaging (MRI) study. The surgical procedure included anterior cervical decompression and fusion (ACF) with the polyetheretherketone (PEEK) cages (Solis™ AS Spacer, Stryker, USA) or structural allograft, and the anterior cervical plate system (ZEPHIR™, Medtronic Sofamor Danek, USA) followed by posterior EOLP with fixation by the titanium miniplates (AO/ASIF miniplate system, Synthes, USA), posterior instrumentation (PI) with a lateral mass or pedicle screw-rod system (Synapse Spine System, DePuy Synthes, USA), and lateral mass fusion. The surgical steps of the posterior procedures are described as follows: (1) adjustment of skull traction to keep cervical alignment in relative kyphosis to ease posterior surgery; (2) placement of C2、C7 pedicle screws and C3-6 lateral mass screws; (3) creation of a hinge side and opening side gutters for EOLP; (4) release of the skull traction and application of a rod in a lordotic posture ipsilateral to the hinge side; (5) elevation of the laminae and fixation of titanium miniplates at the opening side; (6) application of a rod ipsilateral to the opening side; (7) chipped bone grafting over the laminae at the hinge side and bilateral lateral mass. Intraoperative neuromonitoring of somatosensory evoked potential was conducted throughout the procedure. All patients wore a hard cervical collar for 12 weeks after surgery.

In this study, we applied the Nurick score, Japanese Orthopedic Association (JOA) score, neck disability index (NDI), and visual analog scale (VAS) of neck pain to evaluate clinical outcomes. The first two scoring systems represented a neurologic deficit status [8, 9], and the last two were used for evaluating functional status [10]. We collected the functional scores preoperatively and postoperatively, 12 and 24 months later. Radiographic outcomes were evaluated as the change of cervical curvature (C2 lower end plate–C7 upper end plate) from neutral lateral plain films, and the range of motion (ROM) was evaluated on the basis of dynamic lateral films preoperatively and postoperatively, 12 and 24 months later [11]. We used computed tomography (CT) scans 6 months postoperatively to assess the bony fusion status. MRI was performed 12 months postoperatively to evaluate the status of decompression.

The SPSS software package, Version 17.0, was used for statistical analysis. To assess statistical significance, an unpaired Student’s *t* test was performed. The level of statistical significance was set as *P* < 0.05.

## Results

The demographic data are shown in Table 1. The study group consisted of 10 women and 10 men. The mean age at the time of surgery was 67.6 ± 15.2 years. The follow-up duration was at least 24 months. Of the patients, eight were smokers, six were diagnosed with type II diabetes mellitus (DM), and 14 were diagnosed with osteoporosis with a bone mineral density T score <−2.5. The mean operation time was 314.5 ± 51.2 min, and the average blood loss was 442.9 ± 236.0 cm^3^. The mean length of hospital stay was 7.1 ± 3.2 days. Most of the patients received ACF for three motion segments and PI for five levels. Four of the patients received laminoplasty for three levels, 14 received laminoplasty for four levels, and two received laminoplasty for five levels.

**Table 1:** Demographic, comorbidity data, and the fusion levels (*N* = 20)

Items	*N* (%)
Age (y/o) (mean ± SD)	67.6 ± 15.2
Gender	
Male	10 (50)
Female	10 (50)
Smoking	
Yes	8 (40)
No	12 (60)
DM	
Yes	6 (30)
No	14 (70)
Osteoporosis	
Yes	14 (70)
No	6 (30)
ACF level	
3	16 (80)
2	4 (20)
PI level	
5 (C3-7)	2 (10)
6 (C2-7)	18 (90)
LP level	
3	4 (20)
4	14 (70)
5	2 (10)

*DM* diabetes mellitus, *ACF* anterior cervical fusion, *PI* posterior instrumentation, *LP* laminoplasty

The clinical and radiographic outcomes evaluated at 12 and 24 months postoperatively were similar; therefore, we show only the results of a comparison between the preoperative and 24-month postoperative data (Table 2). The JOA score improved from 10.1 ± 1.6 to 15.7 ± 1.8. The Nurick score decreased from 2.6 ± 0.7 to 0.4 ± 0.9, and the NDI decreased from 39.9 ± 6.1 to 20.4 ± 3.2. The mean JOA recovery rate was 72.3 ± 10.5 %. The cervical curvature improved from a 5.0 ± 3.7° kyphotic curve to a 9.3 ± 2.1° lordotic curve. The mean ROM decreased from 15.4 ± 5.4° to 1.4 ± 0.4°. The C2-7 ROM of all patients was close to 0°.

**Table 2:** Preoperative and postoperative functional and radiographic outcomes status and their correlation (*N* = 20)

Items	Preop	Postop	*T* value	*P* value
Functional outcome				
JOA score	10.1 ± 1.6	15.7 ± 1.8	−27.58	<0.001*
Nurick score	2.6 ± 0.7	0.4 ± 0.9	15.00	0.001*
NDI	39.9 ± 6.1	22.4 ± 3.8	−16.63	0.001*
Neck pain VAS	6.2 ± 0.8	2.0 ± 1.3	13.28	0.118
Radiographic outcome				
Cervical curvature (degree)	−5.0 ± 3.7	9.3 ± 2.1	−8.67	<0.001*
ROM (degree)	15.4 ± 5.4	1.0 ± 0.4	9.81	0.311

Data are presented as mean ± standard deviation

*JOA* Japanese Orthopedic Association, *NDI* neck disability index, *VAS* visual analog scale, *ROM* range of motion

*Means *P* value <0.05

During the follow-up period, one patient exhibited a superficial posterior wound infection and recovered after 2 weeks of antibiotics treatment. There were no cases of pseudarthrosis or hardware loosening. All patients exhibited favorable union according to CT scans that were performed 6 months postoperatively, and there was no recurrence of spinal cord compression caused by closure of the opened laminae, according to the MRI study that was conducted 12 months postoperatively.

**Fig. 1:**
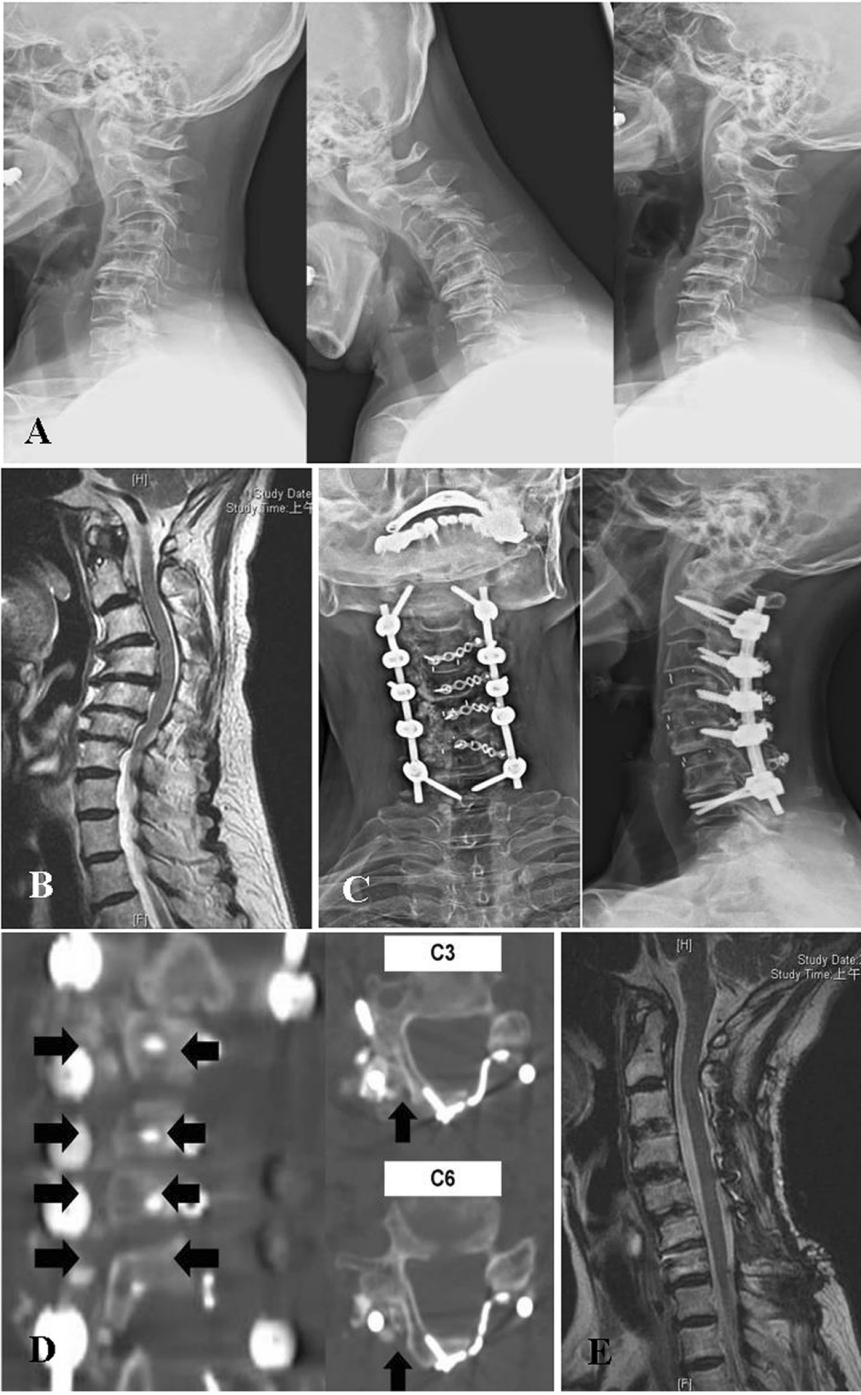
A 55-year-old woman underwent C3-6 anterior diskectomy with fusion, posterior instrumented fusion with C2, C7 pedicle screws and C3-5 lateral mass screws, and posterior decompression with C3-6 laminoplasty. **a** Preoperative X-rays show fixed cervical kyphosis with C2-7 marked spondylosis. **b** Preoperative midsagittal T2-weighted magnetic resonance imaging shows that C2-7 stenosis with spinal cord compression. **c** Postoperative X-rays show that cervical curvature has been restored and the fusion structure is solid. **d** Postoperative 6 months computed tomography scans demonstrate that hinge side fusion bed union (*black arrows*). **e** Postoperative 12 months midsagittal T2-weighted magnetic resonance imaging shows patent spinal cord without compression

*Case report*. A 75-year-old woman had comorbid hypertension, type II DM, and osteoporosis under regular medication control. Bilateral hand clumsiness, progressive unsteady gait with bilateral thigh weakness, and aggravated neck pain were noted in preoperative 1 month. She visited our outpatient department for help, and a positive long-tract sign and Spurling sign were identified. Preoperative plain films revealed fixed cervical kyphosis (Fig. 1a), and MRI revealed C2-7 stenosis with spinal cord compression (Fig. 1b). She received C3-6 ACF, PI with C2, seven pedicle screws and C3-5 lateral mass screws, and posterior decompression (PD) with C3-6 EOLP (Fig. 1c). The bone graft was placed over the laminae of the hinge side and bilateral lateral mass. The symptoms significantly improved after the operation, and a postoperative rehabilitation program was arranged. The JOA score improved from 10 to 16, the Nurick score decreased from 3 to 1, and the NDI decreased from 40 to 20. The cervical curvature improved from a 5° kyphotic curve to an 8° lordotic curve. A CT scan taken 6 months postoperatively revealed solid union over posterior as well as anterior fusion sites (Fig. 1d). Postoperative MRI after 12 months showed a patent spinal cord (Fig. 1e).

## Discussion

In this study, 20 patients received anterior cervical fusion and posterior instrumented fusion with decompression through laminoplasty. The clinical outcomes and cervical spine alignment showed significant improvement without complications of pseudarthrosis or implant failure that may require revision operations. DCKS is an indication for circumferential fusion with decompression [3]. Through an anterior approach, we initially restored the cervical lordosis and disk height; subsequently, posterior stabilization provided a tension band force to retain the alignment. Under the stable structure of a reconstructed cervical spine, the other essential factor of improved surgical outcomes is adequate decompression. Laminectomy is most commonly used in this type of reconstructive surgery, but it leads to significant disfiguring of cervical spine due to muscle atrophy and loss of lamina and the complication of perineural adhesion that can not only cause tethering of the nerve to the surrounding tissue and chronic persistent neurologic symptoms [12, 13] but also increase the difficulty of revision surgeries. Laminoplasty can prevent this problem through the preservation of bony elements. The other advantage of laminoplasty is that it provides a larger fusion bed for bone grafting, which is a crucial factor for high fusion quality, in addition to anterior fusion and PI, especially for patients with old age and comorbidities, which are known causes of delayed bone union and nonunion [14–16]. Solid posterior stabilization with high fusion quality can provide strong support for long-segment anterior fusion indicated for correcting fixed kyphosis and creating solid fusion mass [17]. In our series, although approximately 50 % of the patients were elderly and had comorbid DM, smoking, and osteoporosis, they still exhibited a high postoperative functional recovery and favorable union after 6 months. Favorable functional results and adequate stability without hardware loosening or pseudarthrosis were postoperatively achieved in these 20 patients after 24 months. This procedure seems to benefit the patients with a high risk of fusion failure or revision surgeries.

The functional outcomes showed significant improvement postoperatively. The mean JOA recovery rate was 72.3 %. The improvement of neurologic function was comparable to other studies of long-level cervical instrumented fusion with laminectomy for DCKS [2, 18, 19]. MRI at postoperative 12 months showed no residual compression of spinal cord. Neck pain VAS decreasing from moderate or severe grade to mild grade and postoperative C2-7 ROM at 24 months approximate 0° revealed good stability of fusion construct. Cervical CT performed at postoperative 6 months showed solid fusion mass over the hinge side of laminoplasty. Postoperative cervical curvature improved from 5.0° kyphosis to 9.3° lordosis. It demonstrated good correction of cervical alignment without collapse. The average operation time and blood loss of the surgical procedure in this study were comparable with those of previous two studies on surgical outcomes of circumferential surgery with laminectomy (Table 3) [2, 20].

**Table 3:** Comparison of blood loss and operation time between studies

Items	Mean age	Diagnosis	ACF	PIF + PD	One stage of ACF and PIF + PD
Present study	67.6 y/o	Degenerative cervical kyphosis with stenosis	ABL: 135.2 ± 85.2 ml	ABL: 307.7 ± 92.3 ml	ABL: 442.9 ± 236.0 ml
			MST: 136.8 ± 52.3 min	MST: 139.3 ± 50.4 min	MST: 314.5 ± 51.2 min
Du et al. [2]	59.6 y/o	CSM/OPLL/cervical stenosis	None	ABL: 650 mL MST: 150 min	None
Epstein et al. [20]	54.0 y/o	OPLL	None	None	ABL: 500 ml
					MST: 522 min

*CSM* cervical spondylotic myelopathy, *OPLL* ossification of the posterior longitudinal ligament, *ABL* average blood loss, *MST* mean surgical time, *ACF* anterior cervical fusion, *PIF + PD* posterior instrumented fusion with decompression

Being aware of several technical points is crucial before performing this complex surgery. With the small field of the posterior cervical spine, correct surgical steps involving PI and EOLP with miniplate fixation and bone grafting are essential factors in reducing the operation time and complication rate. Through the method we applied in this study, the advantages of these posterior procedures were combined effectively without problems. In addition, a one-stage anterior and posterior surgery for DCKS can enable achieving superior surgical outcomes through ACF, primarily to correct the kyphotic curve and sequential posterior instrumented fusion with decompression to keep the lordotic curve and decompress the spinal cord. However, placing a patient with an endangered spinal cord in a relative lordotic supine position to perform the anterior surgery would cause neurologic deterioration. Therefore, patients should be asked to extend their neck consciously before the surgery to determine whether a neurologic compromise occurs. If a neurologic compromise occurs during neck extension, then more complex posterior anterior posterior surgical steps should be considered. For patients with DCKS, continuous intraoperative neuromonitoring should be performed.

This study had limitations. The number of patients was too low and the follow-up period was short. Furthermore, we did not evaluate the biomechanical effect on and contribution of each technique to the circumferential cervical surgery. Further study is required to compare circumferential cervical surgery with laminoplasty or laminectomy in cases of identical operative indications.

## Conclusion

The surgical aims for DCKS are adequate decompression, solid stabilization with fusion, and correction of kyphosis to at least the neutral position [21]. EOLP applied in cervical fusion as a decompression method may improve functional outcomes and reduce the complications of laminectomy by preventing perineural adhesion and providing an additional fusion bed at the hinge side. These advantages seem to benefit the patients who suffered from DCKS.
